# P-1054. BTK drives neutrophil activation for sterilizing antifungal immunity

**DOI:** 10.1093/ofid/ofae631.1243

**Published:** 2025-01-29

**Authors:** Marissa Agathonia Zarakas, Jigar Desai, Andrew Wishart, Cristina Cunha, Agostinho Carvalho, Tobias M Hohl, Michail S Lionakis

**Affiliations:** UPMC, Pittsburgh, Pennsylvania; Center for Discovery & Innovation, Hackensack Meridian Health, Nutley, New Jersey; National Institutes of Health, Bethesda, Maryland; University of Minho, Braga, Braga, Portugal; UMinho, Braga, Braga, Portugal; Memorial Sloan Kettering, New York, New York; National Institute of Allergy and Infectious Diseases (NIAID)/National Institutes of Health (NIH), Bethesda, MD

## Abstract

**Background:**

Bruton’s tyrosine kinase (BTK) is a critical component of B-cell receptor signaling and regulates B-cell proliferation and survival. Ibrutinib is an irreversible inhibitor of BTK and is successfully used in treatment of various B-cell malignancies. However, it has been associated with increased risk for invasive aspergillosis (IA).

Model of BTK-dependent responses promoting neutrophil activation in response to Aspergillus fumigatus.

**Figure:**
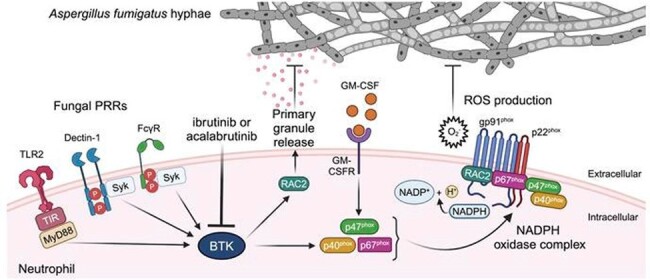

**Methods:**

To shed light in the susceptibility of ibrutinib-treated patients, we studied BTK-dependent fungal responses in neutrophils from diverse populations, including healthy donors, BTKi-treated patients, and X-linked agammaglobulinemia patients.

**Results:**

In response to fungal exposure, BTK was activated in human neutrophils in a TLR2-, Dectin-1-, and FcγR-dependent manner, leading to oxidative burst. BTK inhibition selectively impaired neutrophils’ capacity to damage *Aspergillus* hyphae, release primary granules and induce oxidative burst by impeding the activation of NADPH oxidase subunit p40^phox^ and GTPase RAC2. Similarly, we found impaired neutrophil function in *Aspergillus*-infected, neutrophil-specific *Btk* deficient mice, whereas neutrophil recruitment and survival were spared. These defects were partially mitigated by GM-CSF via enhancing p47^phox^ activation, supporting a role for GM-CSF use in susceptible patients. The presence of the *BTK* rs5951308 (T >C; E24G) in donors of allogeneic hematopoietic transplant recipients was independently associated with increased risk of IA.

**Conclusion:**

Our data uncover a previously unappreciated role for BTK in fungal immune surveillance against *Aspergillus fumigatus*.

**Disclosures:**

**All Authors**: No reported disclosures

